# Supplementary figures and images for: The Establishment and Spread of a Newly Introduced Begomovirus in a Dry Tropical Environment Using Tomato Yellow Leaf Curl Virus as a Case Study
Source: Plants (Basel). 2022 Mar 14;11(6):776. doi: 10.3390/plants11060776 (PMC8952566; doi:10.3390/plants11060776)

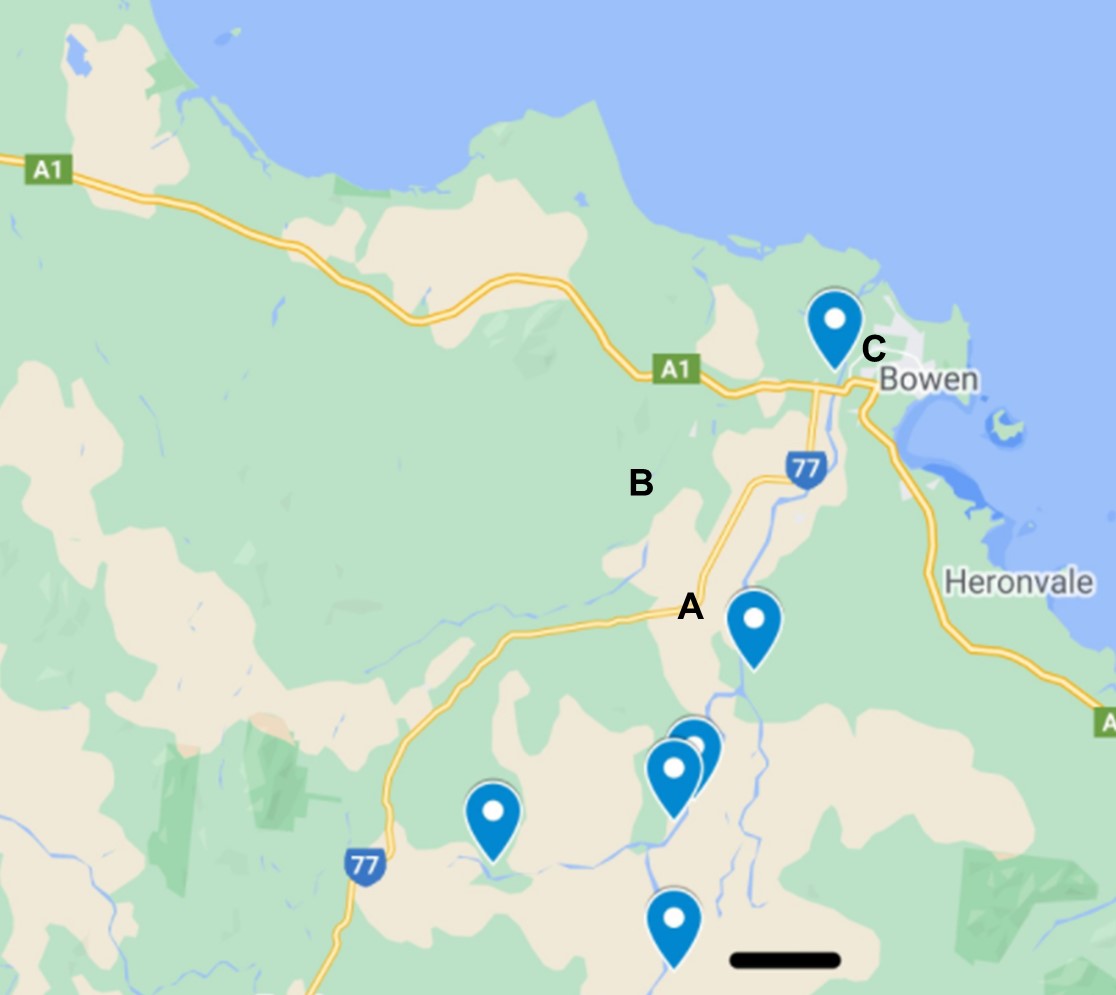

Supplement: Supplementary file 1 [file plants-11-00776-s001.zip › plants-1591945-supplementary.jpg]
